# Endostar combined with radiotherapy increases radiation sensitivity by decreasing the expression of TGF-β1, HIF-1α and bFGF

**DOI:** 10.3892/etm.2014.1526

**Published:** 2014-02-07

**Authors:** YAOGUI WU, YONGFA ZHENG, ZHIXIANG SHEN, WEI GE, YISHAN XIE, CHANGHU LI

**Affiliations:** 1 Department of Oncology, Wuhan University, Renmin Hospital, Wuhan, Hubei 430060, P.R. China; 2 Department of Gastroenterology, Wuhan University, Renmin Hospital, Wuhan, Hubei 430060, P.R. China

**Keywords:** endostar, radiotherapy, tumor angiogenesis, radiation sensitivity

## Abstract

The aim of the present study was to determine how Endostar inhibits tumor angiogenesis and increases radiation sensitivity when combined with radiotherapy. *In vitro* studies were conducted to analyze the expression levels of transforming growth factor-β1 (TGF-β1), hypoxia-inducible factor 1 (HIF-1α) and basic fibroblast growth factor (bFGF) in lung adenocarcinoma A549 cells, using the antiangiogenesis drug Endostar combined with radiotherapy. In addition, lung adenocarcinoma A549 cell apoptosis was detected via Hoechst staining. The combination of Endostar with radiotherapy was investigated and the results indicated that this combination significantly inhibited tumor cell proliferation and TGF-β1, HIF-1α and bFGF expression. Changes in gene expression were found to promote apoptosis, thus, enhancing the inhibition of tumor angiogenesis and ultimately inhibiting tumor cell growth, invasion and metastasis.

## Introduction

Radiation therapy is an important treatment approach for locally advanced non-small cell lung cancer (1). However, tumor recurrence and metastasis are the root causes of radiotherapy failure (2). Numerous studies are aiming to improve the control rate of radiotherapy on tumor (1–3). Folkman (4) identified that blood supplied to tumor blood vessels was significant for solid tumor growth. A tumor with a diameter in excess of 2 mm requires constant provision of nutrients and oxygen from neovascularization for continued growth. Tumor growth may therefore be inhibited via the prevention of tumor angiogenesis (5). Thus, the present study has proposed a novel strategy for tumor antiangiogenesis therapy.

In China, Endostar has been developed (also known as novel recombinant human endostatin), where nine amino acids have been added to the end of the endostatin peptide chain. Endostar has been shown to inhibit migration and induce apoptosis in endothelial cells of new blood vessels. In addition, Endostar exerts an antiangiogenic effect through the adjustment of a variety of signaling pathways that lead to tumor growth, which indirectly results in tumor dormancy or regression (6,7).

Hypoxia inducible factor-1 (HIF-1α) is an important regulatory factor that enables tumor cells to endure a hypoxic microenvironment. HIF-1α promotes tumor angiogenesis and growth by promoting tumor angiogenesis and metabolism-associated gene transcription (8,9). In addition, HIF-1α indirectly reflects the extent of tumor oxygenation.

Carbonic anhydrase IX (CA-IX) is a cell-specific tumor-associated protein which is overexpressed in numerous types of tumors (10). Studies have shown that CA-IX is a factor closely correlated with hypoxia, and it is considered to be a reliable marker of hypoxia (10,11).

Transforming growth factor-β1 (TGF-β1) is a multifunctional regulating peptide that is considered to be closely associated with tumor invasion and metastasis via the degradation of extracellular matrix, the promotion of tumor angiogenesis and the inhibition of the immune system and other channels.

Basic fibroblast growth factor (bFGF) is a predominant angiogenic growth factor in tumor angiogenesis (12). It is a normal micro-substance in human tissues that aids the regulation of cellular DNA synthesis, the promotion of cell division and the stimulation of vascular growth.

In the present study, Endostar was adopted as a vascular specific inhibitor with the aim of investigating whether a synergistic effect exists in its conjunctive use with radiotherapy; possible mechanisms of synergy were also determined.

## Materials and methods

### Cell culture, tumor model and irradiation

The A549 human lung adenocarcinoma cancer cell line (American Type Culture Collection, Manassas, VA, USA) was cultured in RPMI 1640 medium (Hyclone, Thermo Scientific, Logan, UT, USA) supplemented with 100 IU/ml penicillin, 100 mg/ml streptomycin and 10% heat inactivated fetal bovine serum (Hangzhou Sijiqing Biological Engineering Materials Co. Ltd., Hangzhou, China) in a 5% CO_2_ humidified atmosphere at 37°C. Logarithmic-phase cells were collected for use throughout the study.

Cervical dislocation was conducted to sacrifice primary Lewis lung carcinoma-bearing C57 mice (SLAC, Changsha, Hunan, China) were used in this study. The mouse tumors were removed and placed in a homogenizer at a ratio of 1 g tumor sample to 4 ml saline. A cell suspension, with the quantity of living cells >95%, was used for the inoculation of subcutaneous tumors in mice. The left hind armpit of each mouse was inoculated with a 0.2 ml cell suspension.

Tumor-bearing mice were fixed on plate following intraperitoneal injection of anesthetic using 1% chloral hydrate and 6 MeV electron beam irradiation. A radiation dose of 2 Gy was administered daily for five days with a total dose of 10 Gy. The study was conducted in strict accordance with the recommendations in the Guide for the Care and Use of Laboratory Animals of the National Institutes of Health. The animal use protocol was reviewed and approved by the Institutional Animal Care and Use Committee of Renmin Hospital of Wuhan University (Wuhan, China).

### Experimental grouping and treatment

A549 cells at a logarithmic phase were randomly divided into four groups. The blank control group did not undergo cell processing. For the Endostar treatment group (ES), 1 μl Endostar (Simcere Co., Ltd., Nanjing, Jiangsu, China) was added to each well of the 6-well cell culture plates. For the radiotherapy group (RT), a single 2 Gy radiation dose was administered. For the Endostar combined with radiotherapy group (ES + RT), 1 μl Endostar was initially added to each well of the 6-well cell culture plates, which was immediately followed by a 2 Gy radiation dose.

In total, 32 Lewis lung tumor mice were randomly divided into four groups. For the blank control group, each mouse was injected with 0.2 ml normal saline for 14 days. For the ES group, each mouse received a subcutaneous injection of 0.2 ml Endostar for 14 days. For the RT group, the mice were subjected to radiotherapy six days after grouping using Co-60 gamma-ray irradiation. The radiation dose was 3 Gy per day for three days with a total dose of 9 Gy.

### Inhibition of cell growth and proliferation

A549 cells in a logarithmic growth phase were seeded in 96-well culture plates. The number of cells in each well was ~5,000. Following 12 h of incubation, the treatment wells were filled with phosphate-buffered saline. Prior to termination of the culture, 10 μl cell counting kit-8 solution (Dojindo, Kumamoto, Japan) was added to each well. The absorbance of each well was determined using an automatic microplate reader with a 450-nm wavelength.

### Detection of cell apoptosis

A549 cells were stained with 10 mg/ml Hoechst 33258 (Sigma Co., Ltd., St. Louis, MO, USA) at 8, 12 and 24 h following treatment. Apoptotic cells were defined as cells morphologically showing cytoplasmic and nuclear shrinkage and chromatin condensation or fragmentation. At least 400 cells were counted per sample and the percentage of total apoptotic cells was calculated.

### Enzyme-linked immunosorbent assay (ELISA)

Following cell processing, the supernatant of the cells was collected at 0, 6, 12, 18 and 24 h, and specimens were frozen at −20°C. Once thawed, ELISA (Sigma Co., Ltd.) was used to measure the HIF-1α, bFGF and TGF-β1 protein concentrations in the supernatant.

### Quantitative polymerase chain reaction (qPCR)

The following PCR primers were synthesized by Wuhan JinSirui (JinSirui. Ltd., Wuhan, Hubei, China): HomoTGF-β1 forward, 5′-CACGTGGAGCTGTACCAGAA-3′ and reverse, 5′-GAACCCGTTGATGTCCACTT-3′; HIF-1α forward, 5′-TGATGACCAGCAACTTGAGG-3′ and reverse, 5′-TGGGGCATGGTAAAAGAAAG-3′; bFGF forward, 5′-GAGAAGAGCGACCCTCACAT-3′ and reverse, 5′-ACTGCCCAGTTCGTTTCAGT-3′; M-CA-IX forward, 5′-CTCGTGATTCTCGGCTACAACT-3′ and reverse, 5′-ACTGGCTCAGGGCTGCTATC-3′; and e-cadherin forward, 5′-AACGCATTGCCACATACACTC-3′ and reverse, 5′-AGCGATGGCGGCATTGTAG-3′.

Equal quantities of RNA were used for each sample for reverse transcription PCR into cDNA. qPCR was then conducted using SYBR^®^-Green I fluorescent dye (Toyobo Co., Ltd., Osaka, Tokyo). PCR amplification and detection were performed using a LightCycler instrument (ABI7500, Life technologies, NK, USA).

### Statistical analysis

SPSS 13.0 software (SPSS, Inc., Chicago, IL, USA) was used for data analysis. Results are presented as the mean ± SD. The Pearson method was used for correlation analysis of HIF-1α and bFGF expression when following a normal distribution. Numerical values were calculated and subjected to a significance test based on paired or unpaired Student’s t-tests. P<0.05 was considered to indicate a statistically significant difference.

## Results

### Growth of cells

**Figure 1:**
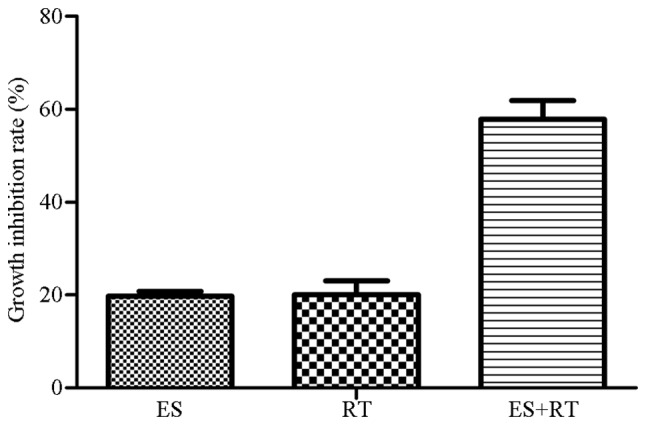
Growth inhibition rate of A549 cells after 24 h in the various groups. Cell proliferation was inhibited in the ES, RT and ES + RT groups. Among these groups, the ES + RT group demonstrated the greatest inhibitory effect and showed a significant difference when compared with the ES and RT groups (P<0.05). Growth inhibition rates for the ES + RT, RT and ES groups were 56.327±5.124, 19.037±5.187 and 19.662±1.536%, respectively. ES, Endostar treatment; RT, radiotherapy; ES + RT, Endostar combined with radiotherapy treatment.

A549 cells were treated for 24 h using various methods, as described in the materials and methods. The ES, RT and ES + RT groups exhibited inhibition of A549 cell growth with the ES + RT group exhibiting the greatest growth inhibition (Fig. 1).

### Detection of apoptosis

**Figure 2:**
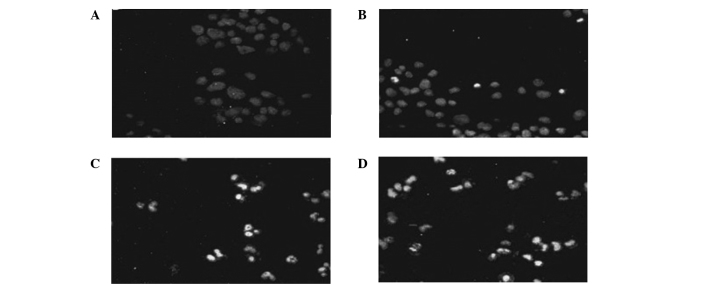
A549 cells in the (A) control, (B) ES, (C) RT and (D) ES + RT groups following treatment with Hoechst staining (magnification, ×400). The lighter regions are apoptotic cells. ES, Endostar treatment; RT, radiotherapy; ES + RT, Endostar combined with radiotherapy treatment.

The ES + RT group exhibited a greater increase in apoptosis compared with the RT and ES groups (P<0.05). The RT group had a greater effect in promoting cell apoptosis compared with the ES group (P<0.05). Compared with the control group, the apoptosis rate of all the other groups increased (Fig. 2).

### HIF-1α, bFGF and TGF-β1 mRNA expression

**Figure 3:**
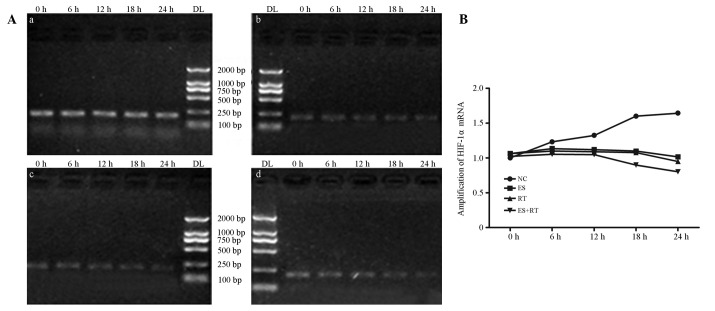
(A) HIF-1α mRNA expression levels in A549 cells in each treatment group. The qPCR electrophoretogram indicates that HIF-1α mRNA expression levels in the ES, RT and ES + RT groups decreased significantly over time when compared with the blank control group. Amplification of HIF-1α mRNA was lowest at 24 h. (B) Amplification of HIF-1α mRNA in each group. HIF-1α mRNA expression levels were statistically significant for each treatment group when compared with the control group (P<0.05). Compared with the RT and ES groups, HIF-1α mRNA amplification of the ES + RT group was statistically significant (P<0.05). HIF-1α, hypoxia-inducible factor 1; NC, control; ES, Endostar treatment; RT, radiotherapy; ES + RT, Endostar combined with radiotherapy treatment; qPCR, quantitative polymerase chain reaction.

**Figure 4:**
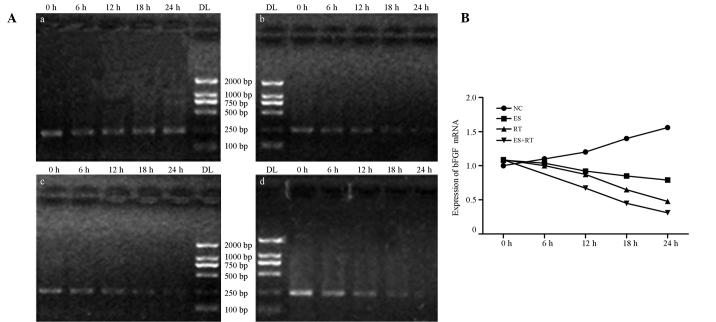
(A) Expression levels of bFGF mRNA in each treatment group. qPCR electrophoresis results indicate that the mRNA expression levels of bFGF in the ES, RT and ES + RT groups decreased over time when compared with the control group. At 24 h, amplification reached a minimum of 0.6387±0.2182, 0.6451±0.1520 and 0.0644±0.0235 in the ES, RT and ES + RT groups, respectively. (B) bFGF mRNA amplification in each group. The decrease in bFGF mRNA expression levels in the ES, RT and ES + RT groups was statistically significant when compared with the control group (P<0.05). The ES + RT group exhibited the lowest bFGF mRNA expression levels; differences were statistically significant when compared with the RT and ES groups (P<0.05). bFGF, basic fibroblast growth factor; NC, control; ES, Endostar treatment; RT, radiotherapy; ES + RT, Endostar combined with radiotherapy treatment; qPCR, quantitative polymerase chain reaction.

**Figure 5:**
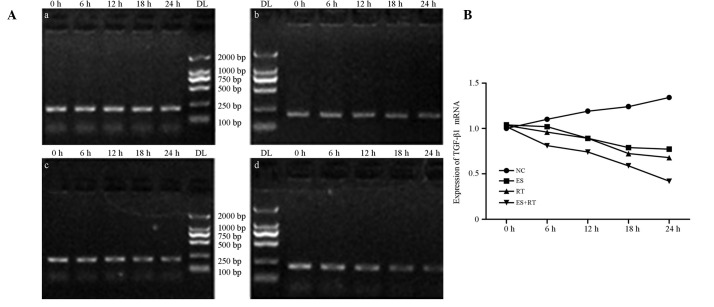
(A) TGF-β1 mRNA expression levels in A549 cells of each group following qPCR amplification, with changes over time (0–24 h) compared with the control group. TGF-β1 expression levels decreased in each treatment group. At 24 h, the difference of the ES + RT group was statistically significant compared with the control and ES groups (P<0.05), but not with the RT group (P>0.05). (B) TGF-β1 mRNA amplification in each group. Results indicated that Endostar combined with radiotherapy significantly inhibited TGF-β1 mRNA expression in A549 cells. TGF-β1, transforming growth factor-β1; NC, control; ES, Endostar treatment; RT, radiotherapy; ES + RT, Endostar combined with radiotherapy treatment; qPCR, quantitative polymerase chain reaction..

A549 cell DNA was amplified using PCR. The mRNA expression levels of TGF-β1, bFGF and HIF-1α changed over time (0–24 h). When compared with the control group, each treatment group exhibited decreased expression. The mRNA expression levels of HIF-1α, bFGF and TGF-β1, compared with the blank control and ES groups, are shown in Figs. 3A, 4A and 5A, respectively. mRNA expression analysis results are shown in Figs. 3B, 4B and 5B. The levels of HIF-1α, bFGF and TGF-β1 in ES + RT group were all significantly decreased when compared with control, ES and RT groups at 24 h (P<0.05). HIF-1α and bFGF expression levels were positively correlated (P<0.01; Figs. 3–5).

### Protein expression of HIF-1α, bFGF and TGF-β1

TGF-β1, HIF-1α and bFGF protein expression levels in the A549 cells of each group exhibited no significant differences prior to processing. However, TGF-β1, HIF-1α and bFGF protein expression levels in A549 cells decreased following treatment with Endostar and/or radiotherapy. Compared with the blank control group, TGF-β1, HIF-1α and bFGF protein expression levels in the RT and ES + RT groups decreased significantly after 24 h (P<0.05).

**Figure 6:**
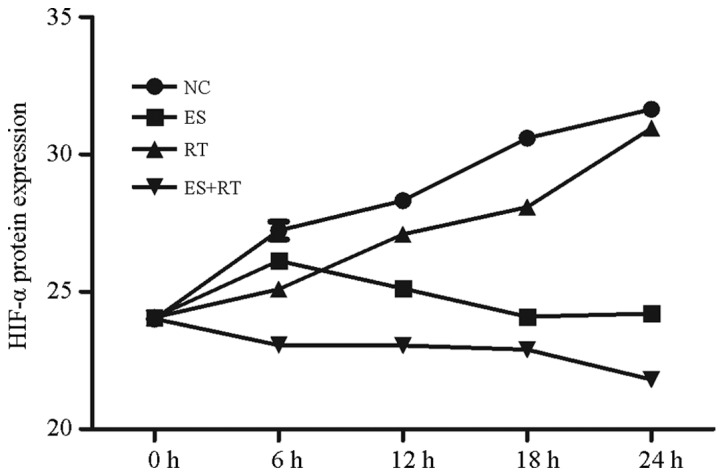
HIF-1α protein expression levels in each group. NC, control; ES, Endostar treatment; RT, radiotherapy; ES + RT, Endostar combined with radiotherapy treatment; HIF-1α, hypoxia-inducible factor 1.

HIF-1α protein expression levels in the supernatants of the ES, RT and ES + RT groups, as measured by ELISA, were shown to decrease increasingly over time. Lowest expression was observed at 24 h (P<0.05; Fig. 6).

**Figure 7:**
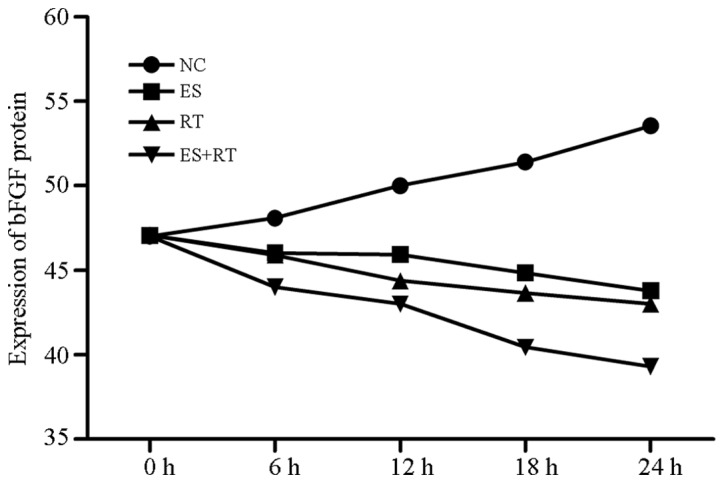
bFGF protein expression levels in each group. NC, control; ES, Endostar treatment; RT, radiotherapy; ES + RT, Endostar combined with radiotherapy treatment; bFGF, basic fibroblast growth factor.

bFGF protein expression levels in the supernatants of the ES, RT and ES +RT groups, as measured by ELISA, were shown to decrease increasingly over time. Lowest expression was observed at 24 h (P<0.05; Fig. 7).

**Figure 8:**
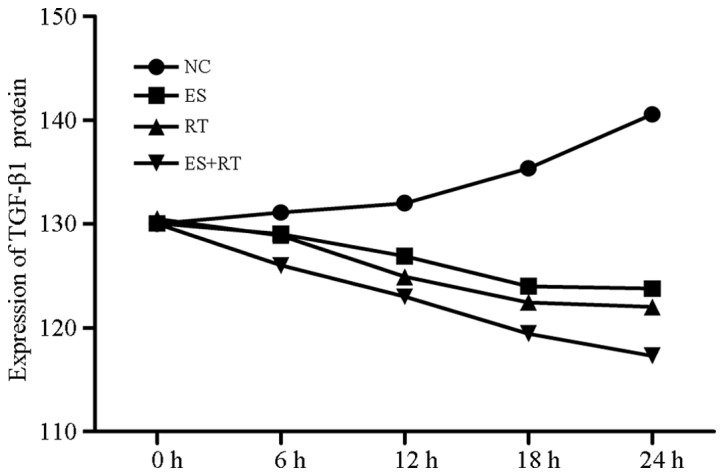
TGF-β1 protein expression levels in each group. NC, control; ES, Endostar treatment; RT, radiotherapy; ES + RT, Endostar combined with radiotherapy treatment; TGF-β1, transforming growth factor-β1

TGF-β1 expression levels in A549 cells also decreased. TGF-β1 expression levels in the ES and ES + RT groups significantly decreased within 24 h. According to statistical analysis, no significant difference was identified in TGF-β1 expression levels among the groups prior to processing (P>0.05; Fig. 8).

### Correlation analysis of HIF-1α and bFGF expression

SPSS 13.0 statistical software was used to analyze the correlation between HIF-1α and bFGF expression. A correlation coefficient of r=−0.80 was derived using the Pearson method (P<0.01). This result demonstrated that HIF-1α and bFGF expression were positively correlated, indicating that increased HIF-1α expression is associated with increased bFGF expression.

## Discussion

Antiangiogenesis treatments block the supply of blood and oxygen to tumors (4,5), inducing a hypoxic state. In the present study, antiangiogenic treatment showed a synergistic effect when combined with radiation therapy. In addition, the present study indicated that antiangiogenic agents target the proliferation of endothelial cells, thus, reducing the oxygen consumption of the tumor and endothelial cells. As a result, oxygenation in tumor microenvironment is improved, as well as the sensitivity to radiotherapy (13). Endostar improves the disordered vascular network of tumors and enables the structure to function under normal vascular status, referred to as the ‘tumor vascular normalization time window’. This ‘normalized vasculature’ improves local tumor blood circulation, reduces tumor interstitial pressure and improves local oxygen partial pressure (13,14). Therefore, Endostar enhances cytotoxic effects on tumor cells within the ‘vascular normalization time window’.

Blood vessels in tumor tissues differ from those in normal vasculature. These poorly distributed and functioned vessels lead to insufficient blood and oxygen supply in tumor tissue (13,14). In the cell studies, tumor cells did not lack oxygen. Radiation resulted in tumor cell apoptosis, however, the extent of apoptosis was increased following Endostar treatment. Cell proliferation in the ES group was inhibited, indicating that Endostar exhibited an antitumor effect.

The results of the present study indicate that radiotherapy combined with Endostar significantly inhibited TGF-β1 and HIF-1α expression. TGF-β1 and HIF-1α serve valuable functions in tumor angiogenesis and vascular remodeling, invasion and metastasis. Hypoxia and nutritional deficiency are the predominant features of solid tumors (15,16). Neovascularization in the tumor tissue is primarily mediated by vascular endothelial growth factor (VEGF) and its signaling pathway. Under hypoxic conditions, VEGF is induced by HIF-1α expression (17). Previous studies have indicated that TGF-β1 and HIF-1α promote angiogenesis through the TGF-β1/PHD2/HIF-1α/VEGF pathway in the process of tumor angiogenesis. A decrease in TGF-β1 reduces the incidence of cell epithelial-mesenchymal transition (18,19); an important biological process involving tumor cell migration and invasion. Inhibition of TGF-β1 overexpression in tumor cells impedes cell invasion and metastasis, thereby improving the efficacy of treatment (20).

In conclusion, Endostar combined with radiotherapy inhibits tumor cell proliferation and the expression of TGF-β1 and HIF-1α, which may affect the expression of VEGF and other genes. VEGF-mediated tumor angiogenesis and TGF-β1-mediated tumor metastasis may be restricted by this therapeutic strategy. The present study provides a theoretical basis for the improvement of treatment in clinical oncology. Improved efficacy may be associated with combined therapy, which significantly downregulates TGF-β1 and HIF-1α expression. Furthermore, combined therapy inhibits tumor cell matrix degradation and reduces cell invasion.

## References

[1] McCloskey P, Balduyck B, Van Schil PE (2013). Radical treatment of non-small cell lung cancer during the last 5 years. Eur J Cancer.

[2] Chi A, Liao Z, Nguyen NP (2010). Systemic review of the patterns of failure following stereotactic body radiation therapy in early-stage non-small-cell lung cancer: clinical implications. Radiother Oncol.

[3] Salama JK, Vokes EE (2013). New radiotherapy and chemoradiotherapy approaches for non-small-cell lung cancer. J Clin Oncol.

[4] Folkman J (1971). Tumor angiogenesis: therapeutic implications. N Engl J Med.

[5] Hanahan D, Weinberg RA (2011). Hallmarks of cancer: the next generation. Cell.

[6] Zhang L, Ge W, Hu K (2012). Endostar down-regulates HIF-1 and VEGF expression and enhances the radioresponse to human lung adenocarcinoma cancer cells. Mol Biol Rep.

[7] Ge W, Cao DD, Wang HM (2011). Endostar combined with chemotherapy versus chemotherapy alone for advanced NSCLCs: a meta-analysis. Asian Pac J Cancer Prev.

[8] Agani F, Jiang BH (2013). Oxygen-independent regulation of HIF-1: novel involvement of PI3K/AKT/mTOR pathway in cancer. Curr Cancer Drug Targets.

[9] Tang CM, Yu J (2013). Hypoxia-inducible factor-1 as a therapeutic target in cancer. J Gastroenterol Hepatol.

[10] Monti SM, Supuran CT, De Simone G (2013). Anticancer carbonic anhydrase inhibitors: a patent review (2008 – 2013). Expert Opin Ther Pat.

[11] Sedlakova O, Svastova E, Takacova M (2014). Carbonic anhydrase IX, a hypoxia-induced catalytic component of the pH regulating machinery in tumors. Front Physiol.

[12] Montesano R, Vassalli JD, Baird A (1986). Basic fibroblast growth factor induces angiogenesis in vitro. Proc Natl Acad Sci USA.

[13] Stylianopoulos T, Jain RK (2013). Combining two strategies to improve perfusion and drug delivery in solid tumors. Proc Natl Acad Sci USA.

[14] Goel S, Duda DG, Xu L (2011). Normalization of the vasculature for treatment of cancer and other diseases. Physiol Rev.

[15] Favaro E, Nardo G, Persano L (2008). Hypoxia inducible factor-1alpha inactivation unveils a link between tumor cell metabolism and hypoxia-induced cell death. Am J Pathol.

[16] Koul HK, Pal M, Koul S (2013). Role of p38 MAP Kinase Signal Transduction in Solid Tumors. Genes Cancer.

[17] Driessen A, Landuyt W, Pastorekova S (2006). Expression of carbonic anhydrase IX (CA IX), a hypoxia-related protein, rather than vascular-endothelial growth factor (VEGF), a pro-angiogenic factor, correlates with an extremely poor prognosis in esophageal and gastric adenocarcinomas. Ann Surg.

[18] Akhurst RJ (2002). TGF-beta antagonists: why suppress a tumor suppressor?. J Clin Invest.

[19] Liang Q, Li L, Zhang J (2013). CDK5 is essential for TGF-β1-induced epithelial-mesenchymal transition and breast cancer progression. Sci Rep.

[20] Chen KC, Chen CY, Lin CJ (2013). Luteolin attenuates TGF-β1-induced epithelial-mesenchymal transition of lung cancer cells by interfering in the PI3K/Akt-NF-κB-Snail pathway. Life Sci.

